# Associations Between Demographic Characteristics, and Diabetes‐Related Knowledge, Attitudes, Practices and Clinical Parameters in People With Diabetes Attending Eye Clinics in Trinidad and Tobago: A Cross‐Sectional Study

**DOI:** 10.1002/hsr2.72948

**Published:** 2026-07-31

**Authors:** Raju Sapkota, Ngozika E. Ezinne, Hugo Onumajuru, Shahina Pardhan

**Affiliations:** ^1^ Vision & Eye Research Institute, School of Medicine, Faculty of Health, Medicine, and Social Care Anglia Ruskin University Cambridge UK; ^2^ Centre for Inclusive Community Eye Health, School of Medicine, Faculty of Health, Medicine, and Social Care Anglia Ruskin University Cambridge UK; ^3^ Optometry Unit, Department of Clinical Surgical Sciences The University of the West Indies St Augustine Trinidad and Tobago; ^4^ Bathurst Rural Clinical School Western Sydney University Sydney New South Wales Australia

**Keywords:** attitude, diabetes, diabetic retinopathy, insulin treatment, knowledge, longer‐term diabetes duration, practice, Trinidad

## Abstract

**Background and Aims:**

Diabetes‐related knowledge, attitudes, and practices (KAP) and associated clinical outcomes, including diabetic retinopathy (DR), have not been adequately examined across patient demographics in Trinidad and Tobago (T&T), which this study examined.

**Methods:**

A total of 194 patients with diabetes (age = 59.56 ± 12.9 years, diabetes duration = 14.29 ± 11.2 years; 59.3% females) attending various eye clinics were recruited via convenience sampling. KAP was assessed using a validated questionnaire. Data on duration of diabetes/treatment, height/weight, blood pressure, fasting blood glucose (FBG) level, and DR status and severity were obtained from recent medical records. Associations between independent (age, gender, education level, employment status) and dependent variables (KAP, clinical data) were explored.

**Results:**

Of the total participants, 43.8% (85/194) were employed, 82.0% (159/194) had above primary‐level education, 51.0% (99/194) were 60 years or older, 49.5% (96/194) reported doing regular physical activity, 60.6% (117/194) would be less concerned at forgetting diabetes medication, and 36.1% (70/194) were unaware that diabetic eye exam differed from basic eye test for spectacles. Females were less willing to discuss their diabetes openly, along with younger participants. DR status/severity was recorded for 78.3% (152/194) using Early Treatment Diabetic Retinopathy Study classification; 42.8% had DR, and 5.7% had sight‐threatening DR (moderate non‐proliferative DR or worse, and/or the presence of diabetic macular edema). FBG (OR = 1.04, 95% CI: 1.01–1.07, *p* = 0.006) and lower (below secondary) level of education (OR = 8.3, 95% CI = 1.53–44.7, *p* = 0.014) predicted DR presence independently.

**Conclusion:**

Notable gaps in diabetes‐related KAP were observed. Women/younger participants were less willing to discuss their diabetes openly. More women knew the importance of physical activity than men. Elevated FBG and lower education levels were independently associated with DR. Our findings support the need for demographically targeted education interventions for diabetic patients attending eye clinics in T&T.

## 1 Introduction

Diabetes mellitus, a chronic metabolic disorder characterized by elevated blood glucose levels, is a major public health problem with an estimated global prevalence of 540 million adult cases in 2021 [1]. Elevated blood glucose levels over a prolonged period can lead to several complications, such as diabetic retinopathy (DR), kidney disease, lower limb amputation, and stroke [2, 3, 4].

Trinidad and Tobago (T&T), a small twin‐island country in the Caribbean, which has a population predominantly of East Indian (40.3%) and African descent (39.6%) [5], has one of the highest prevalence rates of diabetes in the region, affecting 14.8% of the adult population (around 1,50,000 cases) [1]. Diabetes accounts for approximately 13.6% of hospital admissions in T&T, with 0.4% of the gross domestic product spent annually on diabetes‐related hospitalizations [6]. According to the National Eye Survey of T&T, DR accounts for nearly one‐fifth (19.1%) of all blindness among people aged 40 years and older, making it the third leading cause after glaucoma and cataract [7].

Evidence suggests that diabetes patients in T&T generally have a limited understanding of diabetes and its control. For example, a high percentage of patients (58.1%) did not know the importance of controlling glycated hemoglobin (HbA1c) [8], and a significant percentage (55.3%) were living with high HbA1c (≥ 7%) levels [9]. In another study, a significantly higher percentage of individuals who received nutritional counseling (76%) answered diabetes‐related questions correctly than those who never received the counseling (70%) [10]. In another study, although 80% of hospital‐admitted patients received dietary advice from a dietitian, nearly half (49.3%) continued to consume sugar [11], suggesting that dietary advice alone may be insufficient to promote healthy behavioral practices. A study conducted in East Trinidad reported that although patients with diabetes demonstrated good knowledge of the importance of blood glucose monitoring, medication adherence, and foot care, their self‐care practices were suboptimal [12]. Similar findings have been reported by studies in other ethnic groups, in which patients failed to translate their knowledge of diabetes management into improved self‐care practices. For example, a study among Chinese patients with diabetes found that although 72.0% reported knowing that exercise was important in controlling diabetes, only 35.0% of them engaged in regular physical activity [13]. A hospital‐based study in Nepal reported that although nearly all patients recognized the importance of physical activity in managing diabetes, only 27% engaged in regular physical activity [12]. Furthermore, a study from Bhutan reported that diabetes patients often presented late to DR services, resulting in a high proportion of them (42.7%) being diagnosed with DR at the first visit [14].

Patients with diabetes from Caribbean countries are reported to encounter common self‐management challenges such as restricted access to healthcare services, difficulty maintaining healthy behavior, greater trust in herbal remedies than conventional medicines, negative attitudes toward living with diabetes, and a lack of family support [15, 16, 17, 18]. An online survey conducted among healthcare providers in T&T reported that a large majority (96.0%) believed facilities for DR services at the primary care level were inadequate [19], highlighting the need for patient‑centered education interventions for patients with diabetes and DR. However, such programmes remain underdeveloped in T&T [19], possibly because diabetes‐related KAP parameters have not been adequately examined across key demographic groups, including age, gender, education level, and employment status. To address this gap, the present study examined the KAP of patients with diabetes attending an eye clinic in T&T, including associations between KAP and socio‐demographic characteristics as well as clinical parameters such as fasting blood glucose, blood pressure, and DR.

## 2 Methods

### 2.1 Study Design

A cross‐sectional study was conducted between February and September 2024, in accordance with the Strengthening the Reporting of Observational Studies in Epidemiology (STROBE) guidelines [20].

### 2.2 Ethical Consideration

Ethical approval was obtained from the Research Ethics Committee of the University of the West Indies, Saint Augustine, Trinidad and Tobago (CREC‐SA.2526/02/2024) to recruit patients from conveniently selected private eye clinics in Trinidad and Tobago. Information outlining the study's objectives and the rationale for participation was disseminated to these clinics. Clinics that provided consent to facilitate patient recruitment were subsequently visited by a research assistant. Formal requests were made to clinic directors to access medical records and obtain lists of patients diagnosed with diabetes. Identified patients were then contacted via telephone and email to invite them to participate in the study. All participants provided informed consent, and their confidentiality was maintained throughout the study using coded identifiers. The study adhered to the Declaration of Helsinki.

### 2.3 Study Population

The study population comprised individuals diagnosed with diabetes who attended various private eye clinics in T&T.

### 2.4 Sampling method/setting

Convenience Sampling Was Used to Select Eye Clinics and Participants Attending Them.

Sample size: Sample size was calculated using the following standard formula [21]

$$n=\frac{z^{2}\mathrm{pq}}{d^{2}};\;n=\frac{1.96^{2}\times 0.148\times \left(1-0.148\right)}{0.05^{2}}\approx 194,$$

*n* = number of samples

*z* = 1.96 (95% confidence level)

*p* = prevalence rate, 14.8% [1]

*q* = (1‐*p*)

*d* = Precision of the prevalence estimate.

### 2.5 Inclusion/Exclusion Criteria

Participants aged 18 years and above with a clinical diagnosis of diabetes, who self‐reported having no history of psychiatric or mental illness (e.g., dementia, depression), who attended general eye clinics, and who consented to participate in the study were included. Participants were excluded if they were too ill to participate, pregnant, or newly diagnosed (within the past month).

### 2.6 Data Collection Procedure

Data were collected by one research assistant using a semi‐structured questionnaire that had been previously validated [13, 22, 23] and pretested in a sample of 10 participants. Data on medical history including duration of diabetes and type of treatment, along with questionnaire‐based information on diabetes‐related KAP and demographic characteristics were collected (Table 1). Clinical findings (height, weight, and blood pressure) and diagnostic test results, including fasting blood glucose levels and the presence or absence of DR, were obtained from the most recent medical records. Sight‐threatening DR was defined according to the Early Treatment of diabetic retinopathy study (ETDRS) classification [24] as retinopathy severity of moderate non‐proliferative diabetic retinopathy or worse, and/or the presence of diabetic macular edema characterized by retinal thickening within 2‐disc diameters of the center of the macula.

**Table 1:** Summary of independent variables (A), diabetes‐related history (B), and dependent variables, including clinical outcomes (C) and KAP parameters (D).

(A)		
Demographics	Category	Percentage
Gender	Male	40.7% (79/194)
	Female	59.3% (115/194)
Education level	Primary	17.2% (33/192)
	> Primary	82.8% (159/192)
Employment status	Employed	44.0% (85/193)
	Unemployed	56.0% (108/193)
Age group	Below 60 years	48.7% (94/193)
	60 years or above	51.3% (99/193)

(B)		
Duration of diabetes and treatment history	Category	Percentage
Duration of diabetes	≤ 10 years	50.5% (97/192)
	> 10 years	49.5% (95/192)
Treatment for diabetes	Insulin/insulin and tablet	23.2% (45/194)
	Non‐insulin (Tablet, Diet)	76.8% (149/194)
Use of medication for high blood pressure	Yes	60.2% (115/191)
	No	39.8% (76/191)
Use of medication for high cholesterol	Yes	29.0% (56/193)
	No	71.0% (137/193)

(C)	
Clinical variable	Mean ± SD
FBG	140.7 ± 42.5 mg/dL
Systolic BP	136.0 ± 19.8 mmHg
Diastolic BP	84.3 ± 9.1 mmHg
BMI	26.8 ± 5.0 kg/m^2^
DR status:	**Percentage**
Sight‐threatening	5.7% (11/194)
Non‐sight threatening	37.1% (72/194)
Normal (No DR present)	35.6% (69/194)
DR grading not available	21.6% (42/194)
SD = Standard Deviation	

(D)		
Question	Response	Percentage
**Knowledge**		
Is BP or cholesterol control important for diabetes management?	Yes	83.9% (162/193)
	No/not sure	16.1% (31/193)
Is diabetes a serious disease?	Yes	95.9% (186/194)
	No/not sure	4.1% (8/194)
Is diabetes control your responsibility, the doctor's, or a joint responsibility?	Only my or doctor's responsibility	58.5% (113/193)
	Both my and doctor's responsibility	41.5% (80/193)
Is physical activity important in controlling diabetes?	Yes	76.8% (149/194)
	No/not sure	23.2% (45/194)
Is smoking harmful to your	Yes	82.4% (154/187)
diabetes?	No/not sure	17.6% (33/187)
Can diabetes cause blindness?	Yes	94.3% (183/194)
	No/not sure	5.7% (11/194)
Are eye tests for spectacles and diabetes different?	Yes	63.2% (120/190)
	No/not sure	36.8% (70/190)
When should you get your eyes tested for diabetes?	Immediately after diagnosis	63.7% (123/193)
	When sight worsens/after 1 year	36.3% (70/193)
**Attitude**		
Would you worry if you forgot to take your medication?	Yes	39.4% (76/193)
	No/not sure	60.6% (117/193)
Would you be comfortable revealing that you have diabetes?	Yes	65.5% (127/194)
	No/not sure	34.5% (67/194)
Would it be difficult to maintain a healthy diet during festivals?	Yes	45.4% (88/194)
	No/not sure	54.6% (106/194)
Would you go for diabetes eye tests if you are seeing well?	Yes	67.0% (130/194)
	No/not sure	33.0% (64/194)
**Practice**		
Do you engage in regular physical activity?	Yes	49.5% (96/194)
	No/not sure	50.5% (98/194)
Do you smoke?	Yes	9.8% (19/194)
	No	90.2% (175/194)
How often do you check your blood sugar?	At least once within 3 months	94.3% (183/194)
	Less frequently/I don't know	5.7% (11/194)
Have you told your family that you have diabetes?	Yes	99.0% (192/194)
	No	1.0% (2/194)
How many times last month did you forget to take diabetic medication? (Mean ± SD)	1.95 ± 3.1	
How many hours of physical exercise do you do every week? (Mean ± SD)	5.3 ± 6.3	
How often last year did you go to a hospital for suddenly uncontrolled blood sugar? (Mean ± SD)	1.4 ± 0.50	

Abbreviation: SD = Standard Deviation.

Prespecified analyses were any associations between demographic variables and diabetes‐related KAP and clinical data. Subgroup analyses included comparisons of demographics, KAP, and clinical data between those participants who had DR and those who did not. Exploratory analyses included the identification of significant predictor variables of DR. Continuous variables were analyzed using an independent samples *t*‐test. Categorical variables were analyzed using the Pearson chi‐square (χ^2^) test. To identify independent predictors of diabetic DR, multiple binary logistic regression analyses were performed. Guidelines for reporting of statistics were followed [25, 26]. Statistical significance was defined a priori as *α* = 0.05 (two‐tailed). Where multiple comparisons were performed, adjusted significance levels were applied. Statistical Package for Social Sciences (SPSS) software (Version 31) was used to analyze data.

## 3 Results

### 3.1 Demographics Data

The mean age of participants was 59.6 ± 12.9 years. Of the total participants, 59.3% (115/194) were females, and 51.0% (99/194) were above the mean age and were categorized into the 60 years or above age group. All participants had attended at least primary school, and 82% (159/194) reported having completed secondary or tertiary education. Among the total participants, 56.0% (108/194) were unemployed.

Table 1 summarizes data on independent variables (demographics), dependent variables (clinical and KAP), and other diabetes‐related information for all 194 participants. For some participants, responses were incomplete (< 4%), or clinical data were not available.

### 3.2 Clinical Data

Participants presented with high mean FBG levels (mean ± SD = 140.7 ± 42.5 mg/dL), with 54.6% (106/194) having above 126 mg/dL. Nearly 22.0% of participants lacked DR grading, primarily due to an inability to obtain a clear fundus image in the setting of dense cataracts and incomplete/undocumented retinal photography. Among those with DR grading, 5.7% (11/194) had sight‐threatening DR, 37.1% (72/194) had non‐sight‐threatening DR, and 35.6% (69/194) had no DR. Half of the total participants reported having diabetes for more than 10 years, 23.2% (45/194) were on insulin or insulin combined with tablets, and 60.2% (115/191) were using medication for high blood pressure.

### 3.3 KAP Data

When asked about whose responsibility it was to ensure good diabetic control, 41.5% (80/193) reported that it was a joint responsibility of themselves and their doctor, and 58.5% (114/193) thought it was either only theirs or their doctor's responsibility, 36.8% (70/190) were unaware that diabetic eye exam differed from basic eye test for spectacles. However, 83.9% (162/193) of participants recognized the importance of blood pressure or cholesterol control in managing diabetes, and 94.3% (183/194) were aware that diabetes can cause blindness. Regarding diabetes‐related attitude, 33.0% (64/194) of participants reported they would not seek eye test for diabetes as long as they were seeing well, 60.6% (117/194) would not worry if they forgot to take their diabetic medication, 34.5% (67/194) reported they would feel uncomfortable disclosing their diabetes to people other than family members and healthcare providers, and 45.5% (88/194) reported that they find it difficult to maintain a healthy diet during festivals and gatherings. Regarding diabetes‐related practices, 94.3% (183/194) of participants reported checking their blood glucose levels at least once every 3 months, whereas 50.5% (98/194) reported not engaging in regular physical activity. Table 2 shows associations between participants' demographics with clinical and diabetes‐related KAP data.

**Table 2:** Associations between independent and dependent variables, including clinical outcomes (A), as well as diabetes‐related knowledge (B), attitude (C), and practice (D).

(A)												
Demographics	Clinical measurements											
	Category	FBG (mg/dL) (mean ± SD	*p*‐value	Systolic BP (mmHg) (mean ± SD)	*p*‐ value	Diastolic BP (mmHg) (mean ± SD)	*p*‐ value	BMI	*p*‐ value	DR present (%)	DR absent (%)	*p*‐value
Age group	< 60 years	138.8 ± 36.3	0.543	132.1 ± ± 13.8	0.020*	83.2 ± 5.7	0.153	27.9 ± 5.4	0.006*	43.4	56.6	0.009*
	≥ 60 years	142.8 ± 48.2		139.3 ± 23.4		85.2 ± 11.4		25.8 ± 4.3		65.3	34.7	
Gender	Male	141.9 ± 34.3	0.768	136.0 ± 19.9	> 0.99	84.6 ± 9.4	0.749	27.7 ± 5.1	0.060	39.8	58.1	0.330
	Female	139.95 ± 47.4		136.9 ± 19.8		84.1 ± 9.1		26.2 ± 4.8		47.8	52.2	
Education level	Primary	146.8 ± 45.5	0.4051	141.4 ± 26.2	0.100	86.5 ± 12.6	0.100	26.5 ± 4.6	0.690	84	16	0.002*
	> primary	139.5 ± 41.9		134.8 ± 18.0		83.8 ± 8.3		26.9 ± 5.1		48.8	51.2	
Employment status	Employed	138.6 ± 29.8	0.5471	131.3 ± 17.6	0.009*	82.5 ± 7.0	0.028*	27.3 ± 5.0	0.207	41.4	58.6	0.003*
	Unemployed	142.4 ± 51.1		139.2 ± 20.4		85.6 ± 10.4		26.4 ± 5.0		65.9	34.1	
FBG = Fasting Blood Glucose, BP = Blood pressure, BMI= Body Mass Index, SD = Standard Deviation, DR = Diabetic Retinopathy												

(B)																										
Demographics	Category	Is BP or cholesterol control important for diabetes management?			Is diabetes a serious disease?			Is diabetes control yours, the doctor's or a joint responsibility?			Is physical activity important in controlling diabetes?			Is smoking harmful to your diabetes?			Can diabetes cause blindness?			Are eye tests for spectacle and diabetes different?				When should you get your eyes tested?		
		Yes	No/not sure	*p*‐value	Yes	No/not sure	*p*‐value	Only my or doctor's	Both my & doctor's	*p*‐value	Yes	No/not sure	*p*‐value	Yes (%)	No/not sure	*p*‐value	Yes	No/not sure	*p*‐value	Yes	No/not sure	*p*‐value	Immediately after diagnosis		After one year/when sight worsens	*p*‐value
Age group	< 60 years (%)	79.8	20.4	0.169	93.6	6.4	0.161	59.1	40.9	0.884	76.6	23.4	0.99	80.2	19.8	0.566	93.6	6.4	0.763	56.5	43.5	0.069	61.7		38.3	0.654
	≥ 60 years (%)	87.9	12.1		98.0	2.0		57.6	42.4		76.8	23.2		84.2	15.8		94.9	5.1		70.1	29.9		65.3		34.7	
Gender	Male (%)	84.6	15.4	> 0.99	94.9	5.1	0.718	53.2	46.8	0.236	69.6	30.4	0.06	80.8	19.2	0.699	94.9	5.1	> 0.99	63.6	36.4	> 0.99	60.3		39.7	0.447
	Female (%)	83.5	16.5		96.5	3.5		62.3	37.7		81.7	18.3		83.5	16.5		93.9	6.1		62.8	37.2		66.1		33.9	
Education level	Primary (%)	87.5	12.5	0.611	93.9	6.1	0.628	45.5	54.5	0.122	69.7	30.3	0.37	78.1	21.2	0.617	84.8	15.2	0.024*	48.4	51.6	0.103	54.5		45.5	0.321
	> primary (%)	83.0	17.0		96.2	3.8		60.8	39.2		78.0	22.0		82.9	17.1		96.2	3.8		65.6	34.4		65.2		34.8	
Employment status	Employed (%)	83.5	16.5	> 0.99	96.5	3.5	0.737	61.9	38.1	0.46	74.1	25.9	0.461	78.6	21.4	0.250	95.3	4.7	0.758	59.0	41.0	0.364	61.2		38.8	0.545
	Unemployed (%)	84.1	15.9		95.4	4.6		55.6	44.4		78.7	21.3		85.4	14.6		93.5	6.5		66.0	34.0		66.4		33.6	

(C)													
Demographics	Category	Would you worry if you forgot to take your medication?			Would you be comfortable revealing that you have diabetes?			Would it be difficult to maintain a healthy diet during festivals?			Would you go for diabetes eye tests if you are seeing well?		
		Yes	No/not sure	*p*‐value	Yes	No	*p*‐value	Yes	No/not sure	*p*‐value	Yes	No/not sure	*p*‐value
Age group	< 60 years (%)	44.1	55.9	0.185	55.3	44.7	0.006*	51.1	48.9	0.113	70.2	29.8	0.445
	≥ 60 years (%)	34.3	65.7		74.7	25.3		39.4	60.6		64.6	35.4	
Gender	Male (%)	32.1	67.9	0.100	77.2	22.8	0.006*	46.8	53.2	0.770	63.3	36.7	0.437
	Female (%)	44.3	55.7		57.4	42.6		44.3	55.7		69.6	30.4	
Education level	Primary (%)	27.3	72.7	0.169	72.7	27.3	0.423	42.4	57.6	0.848	54.5	45.5	0.154
	> primary (%)	41.8	58.2		64.2	35.8		45.9	54.1		69.2	30.8	
Employment status	Employed (%)	41.7	58.3	0.656	58.8	41.2	0.092	52.9	47.1	0.059	68.2	31.8	0.878
	Unemployed (%)	38.0	62.0		71.3	28.7		38.9	61.1		66.7	33.3	

(D)																			
Demographics	Category	Do you engage in regular physical activity?			Do you smoke?			How often do you check your blood sugar?			Have you told your family that you have diabetes?			How many times last month did you forget to take diabetic medication?		How many hours of physical exercise do you do every week?		How often last year did you go hospital for suddenly uncontrolled blood sugar?	
		Yes	No/not sure	*p*‐value	Yes	No	*p*‐value	At least once in 3 months	Less frequently	*p*‐value	Yes	No/not sure	*p*‐value	(Mean ± SD)	*p*‐value	(Mean ± SD)	*p*‐value	(Mean ± SD)	*p*‐value
Age group	< 60 years	51.1	48.9	0.774	13.8	86.2	0.091	94.7	5.3	> 0.99	98.9	1.1	> 0.99	2.0 ± 3.6	0.849	4.5 ± 7.8	0.021*	1.4 ± 0.5	0.573
	≥ 60 years (%)	48.5	51.5		6.1	93.9		93.9	6.1		99.0	1.0		1.9 ± 2.6		4.2 ± 4.3		1.4 ± 0.5	
Gender	Male (%)	51.9	48.1	0.661	12.7	87.3	0.327	96.2	3.8	0.530	98.7	1.3	> 0.99	2.0 ± 2.6	0.975	6.8 ± 8.0	0.012*	1.4 ± 0.5	0.800
	Female (%)	47.8	52.2		7.8	92.2		93.0	7.0		99.1	0.9		2.0 ± 3.5		4.3 ± 4.8		1.4 ± 0.5	
Education level	Primary (%)	45.5	54.5	0.705	12.1	87.9	0.748	90.9	9.1	0.362	97.0	3.0	0.315	2.0 ± 2.6	0.948	4.3 ± 5.9	0.346	1.5 ± 0.5	0.036*
	> primary (%)	49.7	50.3		9.4	90.6		96.2	3.8		99.4	0.6		2.0 ± 3.3		5.5 ± 6.4		1.3 ± 0.5	
Employment status	Employed (%)	55.3	44.7	0.149	16.5	83.5	0.007*	96.5	3.5	0.352	98.8	1.2	> 0.99	2.0 ± 3.6	0.858	7.0 ± 7.9	0.001*	1.4 ± 0.5	0.931
	Unemployed (%)	44.4	55.6		4.6	95.4		92.6	7.4		99.1	0.9		1.9 ± 2.7		3.9 ± 4.2		1.4 ± 0.5	

Abbreviation: SD = Standard Deviation.

Univariate analysis shows that older age (≥ 60 years) was significantly associated with high blood pressure, *t*(163) = 2.37, *p* = 0.019, effect size = 0.37, 95% CI = 0.06‐0.68 and the presence of DR, *χ* ^2^ (1151) = 7.3, *p* = 0.009, effect size = 0.22. Female sex was significantly associated with doing fewer hours of weekly physical activity (*t*(165) = −2.5, *p* = 0.012, effect size = 0.40, 95% CI = 0.09–0.72) and being more hesitant to talk about their diabetes openly (*χ* ^2^ (1194) = 8.14, *p* = 0.006, effect size = 0.22). Lower education level (up to primary) was significantly associated with a higher percentage of DR (*χ* ^2^ (1152) = 10.43, *p* = 0.002, effect size = 0.26, not knowing that diabetes can cause blindness (χ^2^ (1152) = 6.55, *p* = 0.024, effect size = 0.18), and experiencing a greater number of episodes of uncontrolled diabetes needing hospital visits, *t*(190) = 2.12, *p* = 0.036, effect size = 0.40, 95% CI = 0.03–0.8). Being unemployed was significantly associated with higher levels of systolic (*t*(163) = 2.64, *p* = 0.009, effect size = 0.41, 95% CI = 0.10‐0.72) and diastolic blood pressure (*t*(163) = 2.2, *p* = 0.028, effect size = 0.35, 95% CI = 0.04–0.66), and doing fewer number of hours of weekly physical activity (*t*(164) = 3.3, *p* = 0.001, effect size= 0.51, 95% CI = 0.20–0.82).

Table 3 presents a comparison of demographics, KAP, and clinical data with the outcome variable of DR, classified as present or absent.

**Table 3:** Comparison of demographics (A), clinical data (B), and diabetes‐related KAP (C) between those with DR present and absent.

(A)				
Demographics	Category	DR		
		% Present	% Absent	*p*‐value
Gender	Male	39.8% (33/83)	47.8% (33/69)	0.330
	Female	60.2% (50/83)	52.2% (36/69)	
Education level	Up to primary	25.3% (21/83)	5.8% (4/69)	0.002*
	> Primary	74.7% (62/83)	94.2% (65/69)	
Employment status	Employed/self‐employed	34.9% (29/83)	59.4% (41/69)	0.003*
	Unemployed	65.1% (54/83)	40.6% (28/69)	
Age group	< 60 years	40.2% (33/82)	62.3% (49/82)	0.009*
	≥ 60 years	59.8% (49/82)	37.7% (33/82)	

(B)			
Variable	DR		
	Present (Mean ± SD)	Absent (Mean ± SD)	*p*‐value
FBG	142.5 ± 31.1 mg/dL	125.7 ± 17.6 mmHg	< 0.001*
Systolic BP	134.3 ± 11.2 mmHg	129.7 ± 14.6 mmHg	0.050*
Diastolic BP	84.4 ± 5.7 mmHg	81.5 ± 6.0 mmHg	0.003*
BMI	27.4 ± 5.6 kg/m2	26.1 ± 3.8 kg/m2	0.095

(C)					
Knowledge:	Participant response	DR present (%)	DR present (%)	*p*‐value	
Is BP or cholesterol control important for diabetes management?	Yes	89	84.1	0.471	
	No/not sure	11.0	15.9		
Is diabetes a serious disease?	Yes	92.8	98.6	0.128	
	No/not sure	7.2	1.4		
Is diabetes control your responsibility, the doctor's or a joint responsibility?	Only mine or my doctor's responsibility	53.7	60.9	0.412	
	Both mine & my doctor's	46.3	39.1		
Is physical activity important in controlling diabetes?	Yes	33.7	58	0.003*	
	No/not sure	66.3	42		
Is smoking harmful to your diabetes?	Yes	85.4	80.6	0.511	
	No/not sure	14.6	19.4		
Can diabetes cause blindness?	Yes	91.6	94.2	0.755	
	No/not sure	8.4	5.8		
Are eye tests for spectacles and diabetes different?	Yes	58.5	64.2	0.504	
	No/not sure	41.5	35.8		
When should you get your eyes tested for diabetes?	Immediately after diagnosis	65.1	72.5	0.382	
	After 1 year/when sight worsens	34.9	27.5		
**Attitude:**					
Would you worry if you forgot to take your medication?	Yes	39.8	39.7	> 0.99	
	No/not sure	60.2	60.3		
Would you be comfortable revealing that you have diabetes?	Yes	63.9	69.6	0.494	
	No/not sure	36.1	30.4		
Would it be difficult to maintain a healthy diet during festivals?	Yes	43.4	36.2	0.409	
	No/not sure	56.6	63.8		
Would you go for diabetes eye tests if you are seeing well????well?	Yes	66.3	66.7	> 0.99	
	No/not sure	33.7	33.3		
**Practice:**					**Practice questions:**
Do you engage in regular physical activity?	Yes	33.7	58	0.003*	
	No/not sure	66.3	42		
Do you smoke?	Yes	13.3	8.7	0.445	
	No	86.7	91.3		
How often do you check your blood sugar?	At least once within 3 months	96.4	97.1	> 0.99	
	Less frequently	3.6	2.9		
Have you told your family that you have got diabetes?	Yes	100.0	97.1	0.204	
	No	0.0	2.9		
How many hours of physical exercise do you do every week? (Mean ± SD)		4.0 ± 5.01	6.2 ± 6.9	0.037*	0.04*
How often last year did you have to attend the hospital for uncontrolled blood sugar?		1.4 ± 0.49	1.4 ± 0.48	0.888	0.89
BP ‐ Blood pressure, BMI ‐ Body Mass index, FBG = Fasting Blood Glucose					

Abbreviations: FBG, Fasting Blood Glucose; BMI, Body Mass index; BP, Blood pressure.

The presence of DR was significantly associated with lower education level ((*χ* ^2^ (1152) = 10.43, *p* = 0.002, effect size = 0.26), unemployment ((*χ* ^2^ (1152) = 9.09, *p* = 0.003, effect size = 0.24), ≥ 60 years age group ((*χ* ^2^ (1151) = 7.3, *p* = 0.003, effect size = 0.24), higher fasting blood glucose (t(146) = 3.9, *p* = 0.001, effect size = 0.65, 95% CI = 0.32–0.98), higher systolic BP (*t*(140) = 3.9, *p* = 0.050, effect size = 0.33, 95% CI = 0.002–0.663, and less number of hours of weekly physical activity (*t*(132) = 2.1, *p* = 0.040, effect size = 0.36, 95% CI = 0.02–0.71), and longer duration of diabetes (*t*(150) = 3.27, *p* = 0.001, effect size = 0.53, 95% CI = 0.21–0.85. A multiple binary logistic regression analysis was conducted to examine the effects of variables identified as significant in the univariate analysis (above) on the presence of DR. The model was statistically significant (*χ* ^2^(7) = 35.2, *p* < 0.001 explaining 34% of variance (Nagelkerke R Square). FBG (OR = 1.04, 95% CI: 1.01–1.07, *p* = 0.006) and below secondary level education (OR = 8.3, 95% CI = 1.53–44.7, *p* = 0.014) were found to be independent predictors of DR.

## 4 Discussion

This study examined diabetes‐related KAP and clinical outcomes, including the presence of DR, across various demographics of patients attending an eye clinic in T&T. It also identified knowledge gaps that would inform the design of demographically targeted interventions aimed at improving diabetes management and reducing the risk of diabetic blindness.

The major findings revealed that a significant proportion of patients (55.3%) had high FBG levels (> 126 mg/dL). While a vast majority of the participants were aware that diabetes is a serious disease (possibly because more than 50% had a diabetic duration of longer than 10 years), 23.2% were still unaware of the importance of engaging in physical activity in managing diabetes. Additionally, 36.1% were unaware that diabetes eye tests differed from routine eye tests for spectacles. This lack of awareness may delay the early detection and management of DR, potentially leading to blindness [27]. The fact that 36.3% of participants were unaware of the importance of undergoing an eye test immediately after a diabetes diagnosis highlights the need to promote early attendance for diabetic eye screening more widely.

Regarding diabetes‐related attitude, a significant proportion of participants (39.4%) said they would not worry about forgetting their medication, which may impact decision‐making linked to diabetes self‐care, and seeking help from healthcare providers [28, 29].

Thirty‐three percent of participants stated that they were not sure if they would attend a diabetic eye appointment if they were seeing well, highlighting the need for educational programs to improve attendance at diabetic eye examinations regardless of their perceived vision. Additionally, the fact that nearly half of the participants found it difficult to maintain a healthy diet during festivals suggests that cultural and social factors significantly influence dietary habits, underscoring the need for culturally sensitive dietary counseling initiatives and peer support

Significant gaps in self‐care practices were observed. For instance, 49.5% of participants did not engage in regular physical activity, despite its well‐established role in good diabetes management [30]. The low levels of participant engagement in physical activity highlight the need for lifestyle modification programs. Nearly a fifth of participants (18.6%) had not undergone an eye examination in the past year. These findings need addressing, as inadequate self‐care practice of not attending diabetic eye examinations is significantly associated with an increased risk of sight‐threatening DR [23, 31, 32].

When the data were examined across different demographics, several key associations were identified. Females were more aware of the role of physical activity in managing diabetes, suggesting that targeted strategies should be developed to increase awareness among men. Younger and female participants were more reluctant to discuss their diabetes outside of healthcare providers and family, which could impact the support they receive from their broader social circles and peers. Employed participants were associated with a higher smoking rate, a major risk factor for DR [33], suggesting that smoking cessation programs would need to be targeted additionally for this group. Furthermore, significant associations found between unemployment and high blood pressure indicated the need for close monitoring of BP in this group. Older participants had higher FBG and blood pressure, suggesting the need for more intensive monitoring of blood glucose and blood pressure in this group.

The percentage of participants with DR in this study was 42.8%, higher than the 19.1% prevalence of DR found by a population‐based study among people 40 years and above in T&T [7]. This is to be expected as data were collected in our study from the eye clinics, and it is expected to be higher as participants would have been referred from the community. Hospital‐based studies from developing countries comparable to T&T, including Bhutan [14] and India [34] have reported DR prevalence rates of 42.2% and 42.5%, respectively, figures which are similar to our findings. A systematic review and meta‐analysis of studies from Latin America and the Caribbean estimated an overall DR prevalence rate of 37.3% among individuals with Type 2 diabetes [35]. In contrast, hospital‐based studies from developed countries such as the USA [36, 37], the UK [38], and Canada [39] have reported lower prevalence rates of DR among diabetic patients, typically below 30.0%.

DR was significantly associated with higher FBG and BP, older age (≥ 60 years), lower education level, unemployment, lack of awareness of the importance of regular physical activity, and lower engagement in physical activity. Our findings highlight the need for targeted education programs to raise awareness of the importance of physical activity and promote its practice, particularly among older adults, the unemployed, individuals with lower educational attainment, and women, to reduce the risk of DR.

Evidence suggests that diabetes self‐management programs that are tailored to the demographic and cultural contexts of individual patients are crucial for the effective control of diabetes and DR [40, 41, 42]. Our study provides important insights in guiding the development of such targeted interventions for diabetic patients in T&T. Our study, however, has some limitations which could not be mitigated. The study was designed to collect data from conveniently sampled hospital/eye clinics; therefore, the generalizability of our findings does not apply to all settings, and a much larger national study is needed. Furthermore, we excluded individuals who were newly diagnosed with diabetes (within the past month) because the period immediately following diagnosis can be associated with heightened emotional stress [43], and we wished to avoid adding further emotional burden to patients due to their recruitment. Also, their treatment plan might not have been finalized yet. Self‐reported data on KAP variables may be subject to recall and social desirability biases. Additionally, while associations between certain KAP parameters and sociodemographic factors were observed, causality cannot be established due to the study's cross‐sectional design.

## 5 Conclusion

Our findings highlight the need to create supportive environments in which open discussions about diabetes are normalized and encouraged, particularly for women and younger individuals. Older participants require more intensive monitoring and management of their diabetes and blood pressure. Future research should focus on developing interventions that address demographic differences influencing positive self‐care practices over time, which is essential for the sustained control of diabetes, and reducing the risk of diabetes‐related blindness among patients in T&T.

## Author Contributions

**Raju Sapkota:** conceptualization, formal analysis, writing – original draft, investigation, writing – review and editing. **Ngozika E. Ezinne:** supervision, writing – review and editing, project administration. **Hugo Onumajuru:** data curation, formal analysis. **Shahina Pardhan:** conceptualization, writing – review and editing, funding acquisition, resources.

## Disclosure

This research was conducted in the absence of any commercial or financial relationships that could be construed as a potential conflict of interest. The supporting source/financial relationships had no involvement in the study design; collection, analysis, and interpretation of data; writing of the report; and the decision to submit the report for publication.

## Ethics Statement

The study protocol was approved by the Research Ethics Committee of the University of the West Indies, Saint Augustine, T&T (CREC‐SA.2526/02/2024).

## Consent

Informed consent was obtained from each participant prior to enrollment.

## Transparency Statement

Dr. Raju Sapkota/Dr. Ezinne Ngozika affirm that this manuscript is an honest, accurate, and transparent account of the study being reported; that no important aspects of the study have been omitted; and that any discrepancies from the study as planned (and, if relevant, registered) have been explained.

## Data Availability

The data that support the findings of this study are available from the corresponding author upon reasonable request.
